# A Standardized Chinese Herbal Decoction, Kai-Xin-San, Restores Decreased Levels of Neurotransmitters and Neurotrophic Factors in the Brain of Chronic Stress-Induced Depressive Rats

**DOI:** 10.1155/2012/149256

**Published:** 2012-08-29

**Authors:** Kevin Yue Zhu, Qing-Qiu Mao, Siu-Po Ip, Roy Chi-Yan Choi, Tina Ting-Xia Dong, David Tai-Wai Lau, Karl Wah-Keung Tsim

**Affiliations:** ^1^Division of Life Science, Center for Chinese Medicine and State Key Laboratory of Molecular Neuroscience, The Hong Kong University of Science and Technology, Clear Water Bay Road, Hong Kong; ^2^School of Chinese Medicine, The Chinese University of Hong Kong, Shatin, N.T., Hong Kong

## Abstract

Kai-xin-san (KXS), a Chinese herbal decoction being prescribed by Sun Simiao in *Beiji Qianjin Yaofang* about 1400 years ago, contains Ginseng Radix et Rhizoma, Polygalae Radix, Acori tatarinowii Rhizoma, and Poria. KXS has been used to treat stress-related psychiatric disease with the symptoms of depression and forgetfulness in ancient China until today. However, the mechanism of its antidepression action is still unknown. Here, the chronic mild-stress-(CMS-) induced depressive rats were applied in exploring the action mechanisms of KXS treatment. Daily intragastric administration of KXS for four weeks significantly alleviated the CMS-induced depressive symptoms displayed by enhanced sucrose consumption. In addition, the expressions of those molecular bio-markers relating to depression in rat brains were altered by the treatment of KXS. These KXS-regulated brain biomarkers included: (i) the levels of dopamine, norepinephrine, and serotonin (ii) the transcript levels of proteins relating to neurotransmitter metabolism; (iii) the transcript levels of neurotrophic factors and their receptors. The results suggested that the anti-depressant-like action of KXS might be mediated by an increase of neurotransmitters and expression of neurotrophic factors and its corresponding receptors in the brain. Thus, KXS could serve as alternative medicine, or health food supplement, for patients suffering from depression.

## 1. Introduction

Today, a lot of people are suffering from a depressive episode. If a person encounters these symptoms, for example, anhedonia (loss of interest and pleasure), persistent depression, difficulty in sleeping, suicidal tendency, occuring together and lasting for more than two weeks without significant improvement, thus major depression disorder is being diagnosed [1]. The major depression disorder (depression) today is a common psychiatric disorder having an incidence up to 15% and perhaps higher for women at 25% [2], which is estimated to be a major burden on mental health service by the year of 2020 according to WHO's prediction. 

 Currently, several therapies are used for the treatment of depression, which can be categorized into two parts, psychotherapy and pharmacotherapy, supplemented with other therapies, for example, electroconvulsive seizures, deep brain stimulation, and exercises [3]. The modulation of neurotransmitters, especially restoring the decreased level in the brain of depressive patients, had become the target for development of anti-depression drugs. At least three categories of antidepression drugs have been developed: (i) the tricyclic anti-depressants, such as imipramine, dothiepin, and clomipramine, (ii) the selective neurotransmitters reuptake inhibitors, such as selective serotonin reuptake inhibitors, and norepinephrine reuptake inhibitors, norepinephrine-dopamine reuptake inhibitors, and (iii) monoamine oxidase inhibitors. However, about 30%–40% of patients are not responding to an initial 4–6-week treatment with these drugs. About 10–15% of patients fail to improve sufficiently, even after several attempts of different treatments, while 12–15% of depressive patients show no response at all, not to mention the possible side effects [4]. Therefore, other theories of the etiology of depression have been proposed, and the deficiency of neurotrophic factors in the brain is an important one [5].

 Traditional Chinese medicine (TCM) has offered a possible therapy for the treatment of depression. The records of treating mental disorders could be found in ancient medicinal books, and a herbal decoction kai-xin-san (KXS) is the most popular one. The first description of KXS is recorded in *Beiji Qianjin Yaofang* <*Thousand Formulae for Emergency*> written by Sun Simiao in Tang Dynasty (i.e., 652 AD). This herbal formula composes of four herbs: Ginseng Radix et Rhizoma (root and rhizome of *Panax ginseng* C. A. Mey.), Polygalae Radix (root of *Polygala tenuifolia* Wild.), Acori Tatarinowii Rhizoma (rhizome of *Acorus tatarinowii* Schott), and Poria (sclerotium of *Poria cocos* (Schw.) Wolf). KXS has been used to treat the diseases having symptoms of depressed mood and morbid forgetfulness [6]. Although this decoction has been used frequently, the mechanism of KXS for anti-depression is still unknown, which therefore hinders the further clinical application of this herbal formula. Here, we applied chronic mild-stress-(CMS-) induced depressive rat models to search the mechanism of KXS on anti-depression. The long-term treatment of a chemical standardized herbal extract of KXS suppressed the CMS-induced depression in rats. In the brains of KXS-treated rats, the levels of neurotransmitters, the mRNA expressions of crucial enzymes in regulating those neurotransmitters, and the mRNA expressions of neurotrophic factors and its receptors were significantly altered.

## 2. Materials and Methods

### 2.1. Chemicals

Dopamine hydrochloride, 5-hydroxyindoleacetic acid, serotonin hydrochloride, and norepinepherine were purchased from Sigma-Aldrich (St. Louis, MO, USA). Internal standards with isotope labeling were 2-(3,4-dihydroxyphenyl)ethyl-1,1,2,2-d_4_-amine HCl (dopamine-d_4_, 98 atom % D); 5-hydroxyindole-4,6,7-d_3_-3-acetic-d_2_-acid (5-hydroxyindole-3-acetic acid-d_5_, 98 atom % D); L-glutamic-2,3,3,4,4-d_5_-acid (glutamate-d_5_, 98 atom % D); serotonin-*α*,*α*,*β*,*β*-d_4_ creatinine sulfate complex (serotonin-d_4_, 98 atom % D); norepinepherine-2,5,6,*α*,*β*,*β*-d_6_ HCl (norepinepherine-d_6_, 98 atom % D). The internal standards were purchased from CDN Isotopes (Quebec, Canada). LC-MS-grade acetonitrile and water were purchased from Capitol Scientific (Austin, TX, USA). Formic acid was purchased from Riedel-de Haën Inc. (Hannover, Germany). Imipramine was purchased from Sigma-Aldrich. RNAzol reagent was purchased from Molecular Research Center (Cincinnati, OH, USA). High-Capacity cDNA Reverse Transcription Kit was purchased from Applied Biosystems (Foster City, CA, USA). KAPA SYBR FAST qPCR Kit was purchased from Kapa Biosystems (Woburn, MA, USA).

### 2.2. Preparation of Extract of KXS Decoction

 KXS decoction composed of the following dried raw materials: 4 g Ginseng Radix et Rhizome (root and rhizome of *P. ginseng*), 4 g Polygalae Radix (root of *P. tenuifolia*), 100 g Acori Tatarinowii Rhizoma (rhizome of *A. tatarinowii*), and 200 g Poria (sclerotium of *P. cocos*). The herbs were purchased from Qingping Market of Chinese herbs in Guangzhou, China, which were authenticated by one of the authors, Dr. Tina T.X. Dong, according to their morphological characteristics. The voucher specimens were deposited in the Centre for Chinese Medicine at Hong Kong University of Science and Technology. The herb materials were combined and boiled in 2,500 mL of water for 2 hours, and the herbs were extracted twice. For the second extraction of KXS, the residue from the first extraction was filtered: the same extraction condition was applied on the filtered residue. Then, the extracts were combined and concentrated to the powder and stored at −80°C. Before the KXS treatment, the powder was re-dissolved and vortexed in room temperature. The herbal extract was chemically standardized as reported previously [7].

### 2.3. Animals and Drug Treatment

 Male Sprague-Dawley rats weighing 200–220 g were obtained from the Laboratory Animal Services Center, Chinese University of Hong Kong. Animals were maintained on a 12-hour light/dark cycle (lights on at 6 : 00 a.m., lights off at 6 : 00 p.m.) under controlled temperature (22 ± 2°C) and humidity (50 ± 10%), and they were given standard diet and water. They were allowed to acclimatize for 7 days before use. The experiments on animals have been approved by the Animal Experimentation Ethics Committee of the Chinese University of Hong Kong and conformed to the guidelines of “Principles of Laboratory Animal Care” (NIH publication no. 80-23, revised 1996). The efforts were made to minimize the number and suffering of animals. Rats were randomly divided into five groups of eight individuals. The control animals were given with saline. For another four groups, the animals were treated simultaneously with chronic mild stress (CMS). The drugs (KXS at 0.9 and 2.7 g/kg/day and imipramine at 20 mg/kg) were intragastrically given daily at 30 min before the stress exposure for the entire 4-week treatment.

### 2.4. Chronic Mild Stress Procedure

**Figure 1:**
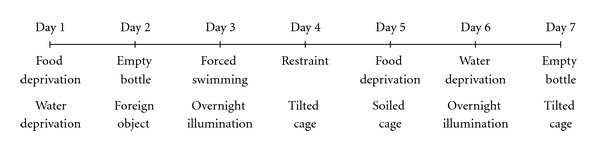
Schedule of chronic mild stress (CMS) procedure. The CMS protocol consisted of the sequential application of a variety of mild stressors. These stressors were randomly scheduled over a one-week period from Day 1 to Day 7 and repeated for 4 weeks during the entire experiment.

 The procedure of CMS was performed as described previously [8]. In brief, the CMS protocol consisted of the sequential application of a variety of mild stressors: (1) food deprivation for 24 hours, (2) water deprivation for 24 hours, (3) exposure to a empty bottle for 1 hours, (4) cage tilt (45°) for 7 hours, (5) overnight illumination, (6) soiled cage (200 mL water in 100 g sawdust bedding) for 24 hours, (7) forced swimming at 8°C for 6 minutes, (8) physically restraint for 2 hours, and (9) exposure to a foreign object (e.g., a piece of plastic) for 24 hours. These stressors were randomly scheduled over a one-week period and repeated throughout the 4 weeks of experiment (see Figure 1). Nonstressed animals were left undisturbed in their home cages except during housekeeping procedures such as cage cleaning.

### 2.5. Rat Sucrose Preference Test

 Sucrose preference test was carried out at the end of 4-week CMS exposure. The test was performed as described previously with minor modifications. Briefly, 72 hours before the test, the rats were trained to adapt to 1% sucrose solution (w/v): two bottles of 1% sucrose solution were placed in each cage, and 24 hours later 1% sucrose in one bottle was replaced with tap water for 24 hours. After the adaptation, rats were deprived of water and food for 24 hours. Sucrose preference test was conducted at 9 : 00 a.m. in which rats were housed in individual cages and were free to access to two bottles containing 100 mL of sucrose solution (1%, w/v) and 100 mL of water, respectively. After 3 hours, the volumes of consumed sucrose solution and water were measured, and the sucrose preference was calculated by the following formula: sucrose preference = sucrose consumption/(water consumption + sucrose consumption) ×100% [8].

### 2.6. Analysis of Neurotransmitters

 The rats were sacrificed by decapitation, and the whole brain tissues were dissected. Brain tissues were rapidly frozen in liquid nitrogen and kept in −80°C for storage. For lysate preparation, the brain tissues were homogenized in ice-cold 0.5 M formic acid with the concentration of 5 mL/g tissue, in the presence of the internal standard at 500 ng/g tissue. The lysates were centrifuged 15,000 ×g for 30 min at 4°C. The supernatant was separated and stored at −20°C until the LC-MS analysis. The detection of neurotransmitters analysis was performed as described [9]. The chromatographic separation was performed on an Agilent UHPLC 1290 series system (Agilent, Waldbronn, Germany), which was equipped with a degasser, a binary pump, an autosampler, and a thermostated column compartment. The brain sample was separated on an ACE C18 column (3.0 *μ*m i.d., 100 mm × 2.1 mm). The mobile phase was composed of 0.1% formic acid in water (A) and 0.1% formic acid in acetonitrile (B) using the following gradient program: 0–2 min, isocratic gradient 1.0% (B); 2–6 min, linear gradient 1.0–90.0% (B); 6–10 min, isocratic gradient 90.0% (B). A preequilibration period of 4 min was used between each run. The flow rate was 0.2 mL/min, the column temperature was 25°C, and the injection volume was 5 *μ*L. An Agilent QQQ-MS/MS (6410A) equipped with an ESI ion source was operated in positive ion mode. The following conditions were optimized: drying gas, nitrogen (10 L/min, 325°C); capillary voltage, 1950 V; scan mode, SRM. The detected ion pairs, the acquired fragmentor, and the collision energy were tuned with the aids of Agilent optimization software (B02.01). The mass spectrometry calibration was performed with the autofeature of Agilent Mass Hunter Chemstation software (version B01.03) using the ESI-L low-concentration tuning mix supplied with the apparatus. Agilent Mass Hunter workstation software version B.01.00 was used for data acquisition and processing.

### 2.7. Total RNA Extraction

 Total RNA from brain tissue was isolated with RNAzol reagent according to the manufacturer's protocol. In details, total brain tissues were added with RNAzol reagent (1.5 mL/g) and homogenized. The homogenate was centrifuged at 16,100 ×g for 5 min at 4°C. The supernatants were removed, added with diethylpyrocarbonate-(DEPC-) treated water (prepared by autoclaving water with DEPC in a 1000 : 1 ratio), and vortexed vigorously for 15 sec, followed by centrifugation at 13,500 rpm (16,100 ×g) for 10 min at 4°C. The aqueous layer was collected and added with half volume of 70% ethanol in DEPC-treated water for RNA precipitation. The RNA pallet was collected by centrifugation at 16,100 ×g for 10 min at 4°C and washed with 70% ethanol in DEPC-treated water twice. After air dry, the RNA was re-suspended in 200 *μ*L of DEPC-treated water. Concentrations of extracted RNA were calculated from the UV absorbance at 260 nm. The quality of RNA was assessed by absorbance at 260 nm and 280 nm, with the ratio of 260/280 nm ranging from 1.90 to 2.10 being acceptable.

### 2.8. Real-Time Quantitative PCR

 Isolated RNAs were reverse transcribed by the moloney murine leukemia virus (MMLV) reverse transcriptase with oligo-d(T) primer in a 20 *μ*L reaction by using High-Capacity cDNA Reverse Transcription Kit. In details, three *μ*g of total RNA was mixed with 1 *μ*L of 0.5 *μ*g/mL oligo-d(T) primer, 1 *μ*L of 10 mM dNTP mix, and RNAase/DNAase-free water in a 12 *μ*L reaction. The mixture was incubated in 65°C for 5 min. Two *μ*L of 0.1 M dithiothreitol (DTT), 1 *μ*L of 40 U/*μ*L RNase out, and 4 *μ*L of 5 × first strand buffer (250 mM Tris-HCl, pH 8.3, 375 mM KCl, 15 mM MgCl_2_) were added into the reaction mix and incubated at 37°C for 5 min. One *μ*L MMLV was added into the reaction and incubated at 37°C for 50 min. Then, the reaction was incubated at 70°C for 15 min. Quantification of the cDNA was determined by UV absorbance at 260 nm and 280 nm by NanoDrop. Applications were performed in an Applied Biosystems PCR system for 40 cycles. Ten *μ*L aliquots of the PCR products were size-separated by electrophoresis on a 2% agarose gel. Real-time quantitative PCR was performed by using SYBR Green Master mix and ROX reference dye, according to the manufacturer's instructions of KAPA SYBR FAST qPCR Kit. In brief, cDNAs were obtained from the reverse transcription of the RNA from rat brain. SYBR green signal was detected by M × 3000 ptm multiplex quantitative PCR machine. Transcript levels were quantified by using the Ct value method [11], where values were normalized by the internal-control GAPDH in the same sample. PCR products were analyzed by gel electrophoresis on a 1.5% agarose gel, and the specificity of amplification was confirmed by the melting curves. Primers employed in RT-PCR and real-time quantitative PCR analyses were listed in Table 1.

## 3. Results

### 3.1. KXS Alleviates the Depressive-Like Symptoms Induced by Chronic Mild Stress

 A chemically standardized herbal extract was prepared according to the ancient recipe: this was an important prerequisite criterion to ensure the repeatability of KXS treatment. KXS was prepared by having Ginseng Radix et Rhizoma, Polygalae Radix, Acori Tatarinowii Rhizoma, and Poria in a ratio of 1 : 1 : 25 : 50. A standardized extraction method of herbal extraction and a quantification of KXS chemically by HPLC-DAD-MS/MS method were developed previously [7]. In addition, an HPLC-MS/MS fingerprint of KXS was shown in Supplementary Figure avalible online at doi:10.1155/2012/149256: this was to ensure the quality of the herbal decoction. By determining the amounts of marker chemicals from each herb, we recommended that a standardized KXS extract should contain no less than 20.4 ± 1.7 mg of ginsenoside Rb_1_, 8.0 ± 0.6 mg of ginsenoside Rd, 19.0 ± 1.3 mg of ginsenoside Re, 24.6 ± 2.2 mg of ginsenoside Rg_1_, 33.6 ± 0.3 mg of 3, 6′-disinapoyl sucrose, 51.4 ± 0.2 mg of *α*-asarone, 1112.4 ± 1.9 mg of *β*-asarone, 21.1 e^−3^ ± 0.4e^−3^ mg of pachymic acid, in 100 g of KXS extract, and the yield of KXS was 14.3 ± 6% (mean ± SD, *n* = 3). These parameters established the chemical standards of KXS for subsequent studies on animals.

**Figure 2:**
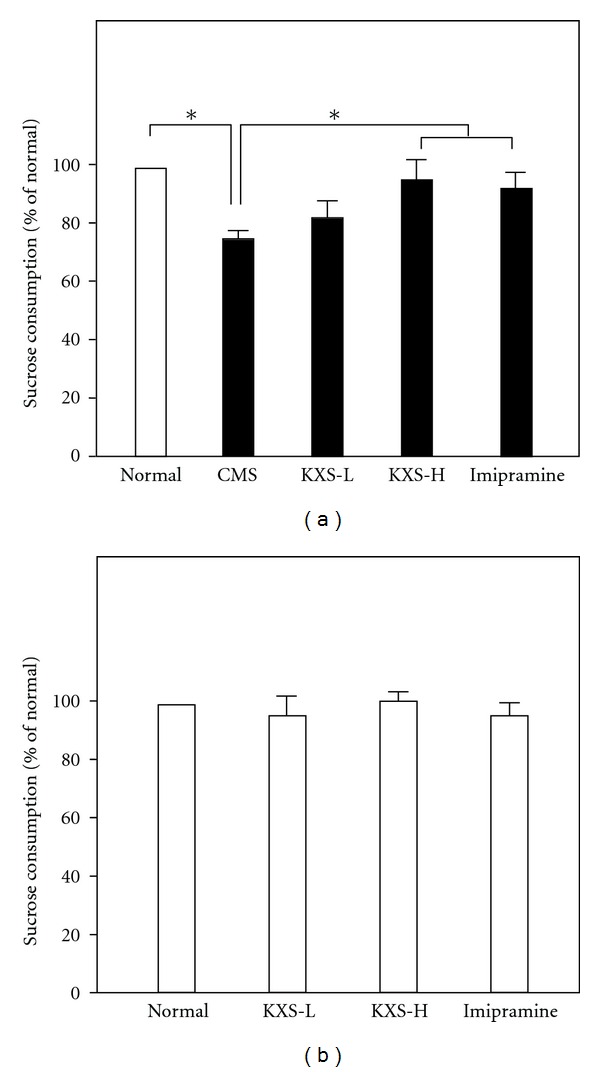
KXS increases the sucrose consumption in CMS-treated rats. (a) Five groups of rats were employed for the sucrose preference test, as stated in Figure 1. KXS treatment, intra-gastrically, was administrated daily at 30 min before the stress exposure for 4 weeks of experimental period. Two doses of KXS were applied including low dosage at 0.9 g/kg (KXS-L) and high dosage at 2.7 g/kg (KXS-H). Imipramine at daily dosage of 20 mg/kg was set as a positive control. (b) Treatment of KXS and imipramine as in (a) but these were all in normal rats. Values are expressed in the percentage of normal (unstressed, or no drug, control), as mean ± SEM (*n* = 8). *P < 0.05.

 To evaluate the anti-depression efficacy of KXS on animal model, the depressive rats induced by CMS were employed for this exploration. The CMS paradigm involved the exposure of animals to a series of mild and unpredictable stressors for 4 weeks (Figure 1). The effect of KXS treatment on the percentage of sucrose consumption in CMS-treated rats was shown in Figure 2(a). Five groups of rats were employed for the sucrose preference test. Imipramine at daily dosage of 20 mg/kg was set as a positive control. KXS treatment was set for two dosages: low dosage (KXS-L) at 0.9 g/kg and high dosage (KXS-H) at 2.7 g/kg. The applied KXS dosage here was estimated according to the current clinical usage in human. A 4-week CMS exposure significantly reduced the percentage of sucrose consumption by ~21% in the animals as compared to the nonstressed control. The long-term treatment of KXS-H increased the sucrose consumption in CMS-treated rats (~20%), as compared to the CMS-treated control, that is, an improved behavior. The treatment of imipramine increased the percentage of sucrose consumption in CMS-treated rats (~18%). The long-term treatment with KXS-L also showed an increase in tendency (~15%), but without statistical significance. Both treatments almost restored the sucrose consumption back to the normal condition. The treatment of KXS, or imipramine, in normal rats however did not show any significant effect on the sucrose consumption (Figure 2(b)). Thus, KXS exerted a profound anti-depression effect in alleviating the depressive-like symptom on CMS-induced depressive rats.

### 3.2. KXS Restores the Decreased Level of Neurotransmitters in Brain of the Depressive Rats

**Figure 3:**
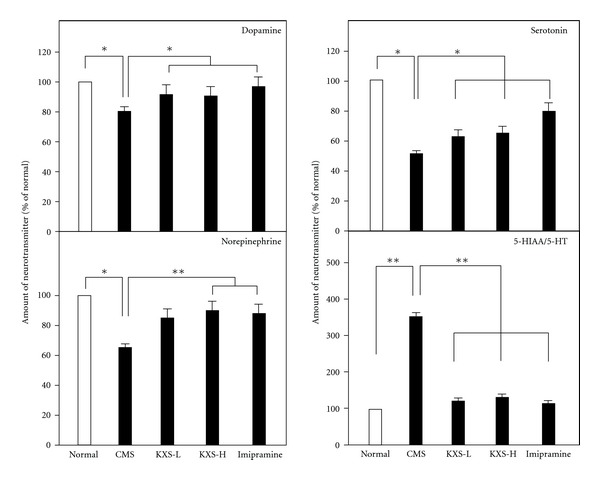
KXS restores the decreased level of neurotransmitters in depressive rats. The treatment of KXS in the rats was as that in Figure 2. The total brain was collected after the treatment. The amounts of dopamine, norepinepherine, serotonin, and 5-HIAA in rat brains were analyzed by LC-MS. Two doses of KXS were applied including low dosage at 0.9 g/kg (KXS-L) and high dosage at 2.7 g/kg (KXS-H). The imipramine at dose of 20 mg/kg was set as the positive control. Values are showed as the mean ± SEM (*n* = 8). *P < 0.05  **P < 0.01.

 The developed HPLC-MS/MS method was employed to determine the total levels of neurotransmitters in the brain of CMS-treated rats [9]. The effects of KXS and imipramine on the amount of neurotransmitters in the brains were summarized in Figure 3. In the CMS-treated rat brains, the reductions of norepinepherine, dopamine, and serotonin were significantly revealed: the decrease was from 20 to 50%. Imipramine treatment was able to fully reverse the effects of CMS on the amounts of norepinepherine, dopamine, and serotonin in the brain. The treatment with KXS, both low and high doses, was effective in reversing the effect of CMS on norepinepherine and dopamine; that is, the levels returned to normal control (Figure 3).

 Here, the amounts of serotonin and 5-hydroxyindoleacetic acid (5-HIAA), a metabolite of serotonin, were analyzed in the KXS-treated rat brains. The ratio of 5-HIAA to serotonin could be used as an index for the turnover of serotonin [12, 13]. The amount of serotonin was markedly reduced in the CMS-treated rat brains (Figure 3). In parallel, the amount of 5-HIAA was markedly increased, that is, an increase of serotonin breakdown. This CMS-induced phenomenon could be significantly reversed by the treatment of KXS, as well as the control treatment of imipramine (Figure 3). The reduced 5-HIAA/5-HT ratio suggested that the turnover of serotonin was decreased by the treatment of KXS.

### 3.3. KXS Regulates the mRNA Expression of Proteins Relating to the Regulation of Neurotransmitters

**Figure 4:**
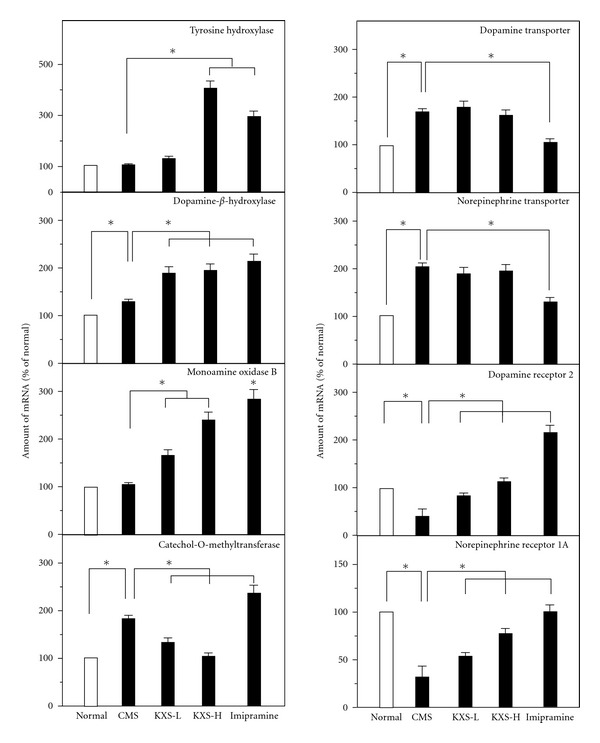
KXS regulates the mRNA expression of proteins relating to catecholamine metabolism in depressive rats. The treatment of KXS in the rats was as that in Figure 2. The total RNA was isolated from the rat brains. The mRNA expression was analyzed by real-time quantitative PCR. Two doses of KXS were applied including low dosage at 0.9 g/kg (KXS-L) and high dosage at 2.7 g/kg (KXS-H). The imipramine at dose of 20 mg/kg was set as the positive control. Values are showed as the mean ± SEM (*n* = 8). *P < 0.05.

 The mRNA levels of proteins relating to the regulation of dopamine and norepinephrine (see Table 1) were determined by quantitative PCR: these proteins included tyrosine hydroxylase, dopamine *β*-hydroxylase, monoamine oxidase B, catechol-O-methyltransferase, dopamine transporter, norepinepherine transporter, dopamine receptor 2, and norepinepherine receptor. The mRNAs encoding enzymes accounting for the metabolism of dopamine and norepinepherine were significantly altered, except tyrosine hydroxylase and monoamine oxidase B, in the CMS-treated rat brains (Figure 4). As compared to CMS control, the KXS treatment could significantly increase the mRNA levels of tyrosine hydroxylase at ~3-fold under KXS-H, dopamine *β*-hydroxylase at ~2-fold under both KXS-L and KXS-H, and monoamine oxidase B from 1.8-fold to 2.5-fold under KXS-L and KXS-H, respectively (Figure 4). In contrast, the level of mRNA encoding catechol-O-methyltransferase was markedly reduced in the KXS-treated depressive rats. Increase of synthesizing enzymes and decrease of degrading enzymes could account for the restoration of dopamine and norepinepherine back to the normal concentration. For the transporters of dopamine and norepinepherine, the mRNA levels of dopamine transporter and norepinepherine transporter were significantly increased in the CMS-treated rat brains (Figure 4). This tendency could be reversed by the imipramine treatment, while the KXS treatment had no effect. For dopamine receptor D2 and norepinepherine receptor 1A (adrenergic receptor *α*1A), CMS could significantly downregulate the mRNA level of the receptors in rat brains (Figure 4). Here, both KXS and imipramine treatments could restore the decreased levels back to the normal condition.

**Figure 5:**
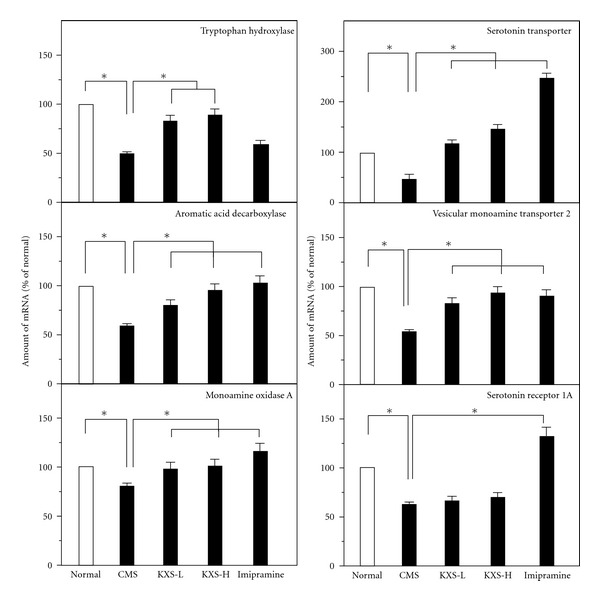
KXS regulates the mRNA expression of proteins relating to serotonin in depressive rats. The treatment of KXS in the rats was as that in Figure 2. The total RNA was isolated from the rat brains. The mRNA expression was analyzed by real-time quantitative PCR. Two doses of KXS were applied including low dosage at 0.9 g/kg (KXS-L) and high dosage at 2.7 g/kg (KXS-H). The imipramine at dose of 20 mg/kg was set as the positive control. Values are showed as the mean ± SEM (*n* = 8). *P < 0.05.

 The mRNA levels of regulating proteins relating to serotonin metabolism (see Table 1) were also determined. The mRNAs encoding tryptophan hydroxylase, aromatic acid decarboxylase and monoamine oxidase A were determined. These mRNAs were significantly reduced under the CMS treatment (Figure 5). The KXS treatment in both dosages restored the mRNA levels of tryptophan hydroxylase, aromatic acid decarboxylase, and monoamine oxidase A. Imipramine could only restored the levels of mRNAs encoding aromatic acid decarboxylase, and monoamine oxidase A (Figure 5). The mRNA expressions of transporter and receptor for serotonin were also determined under the herbal treatment. The CMS-suppressed mRNA levels of these proteins were restore by the treatment of KXS significantly, except serotonin receptor 1A (Figure 5).

### 3.4. Effect of KXS on mRNA Expression Levels of Proteins Related to the Regulation of Neurotrophic Factors

**Figure 6:**
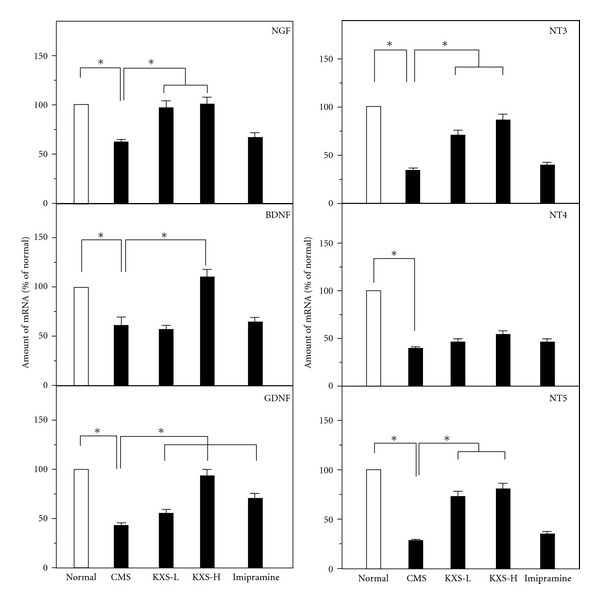
KXS increases the mRNA expression of neurotrophic factors in depressive rats. The treatment of KXS in the rats was as that in Figure 2. The total RNA was isolated from the rat brains. The mRNA expression was analyzed by real-time quantitative PCR. Two doses of KXS were applied including low dosage at 0.9 g/kg (KXS-L) and high dosage at 2.7 g/kg (KXS-H). The imipramine at dose of 20 mg/kg was set as the positive control. Values are showed as the mean ± SEM (*n* = 8). *P < 0.05.

**Figure 7:**
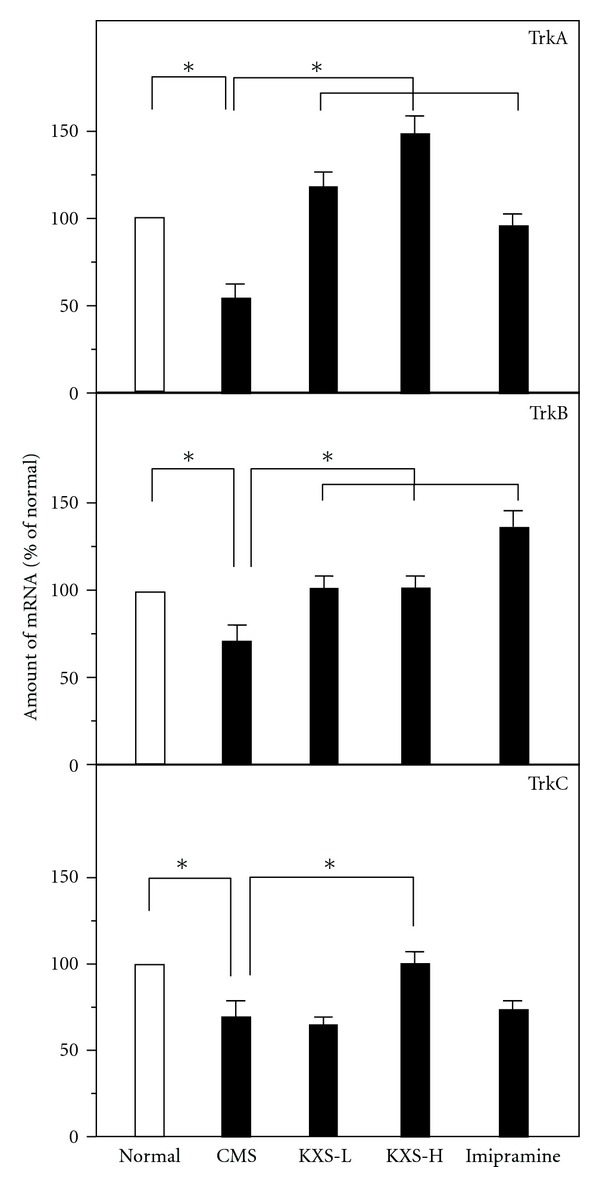
KXS increases the mRNA expression of neurotrophic receptors in depressive rats. The treatment of KXS in the rats was as that in Figure 2. The total RNA was isolated from the rat brains. The mRNA expression was analyzed by real-time quantitative PCR. Two doses of KXS were applied including low dosage at 0.9 g/kg (KXS-L) and high dosage at 2.7 g/kg (KXS-H). The imipramine at dose of 20 mg/kg was set as the positive control. Values are showed as the mean ± SEM (*n* = 8). *P < 0.05.

 The deficiency of neurotrophic factors in the brain was one of the theories in accounting for depression. Here, the expression levels of mRNA encoding NGF, BDNF, GDNF, NT3, NT4, and NT5 and their related receptors were explored. In the CMS-treated rat brains, the mRNA expressions of neurotropic factors were significantly reduced in all cases (Figure 6). The treatment of KXS in CMS-treated rats could increase the expressions of NGF, BDNF, GDNF, NT3, and NT5 mRNAs but not the mRNA of NT4. In contrast, the treatment of imipramine did not show any effect on the CMS-reduced mRNA expression of those neurotropic factors, except GDNF (Figure 6). In addition, the mRNA expressions of Trk A, Trk B, and Trk C receptors were determined: these expressions showed a reduction in the CMS-treated rat brains (Figure 7). In parallel, the levels of Trk A, Trk B and Trk C receptors in the CMS-treated rat brains were restored under the treatment of KXS. Imipramine treatment could only restore the CMS-suppressed Trk A and Trk B receptors (Figure 7).

## 4. Discussions

 KXS, an ancient Chinese herbal decoction, has been used in Chinese medicinal herbal mixture in treating anti-depression; however, the action mechanism of which in brain functions has not been revealed. Here, we provided different lines of evidence to support the anti-depression role of KXS in CMS rat model system. The intake of KXS in the CMS-treated rats could result in the following: (i) the sucrose consumption was increased; (ii) the amounts of dopamine, norepinephrine, and serotonin, as well as its metabolic proteins including the transporters and receptors, were regulated; (iii) the amounts of NGF, BDNF, GDNF, NT3, NT4, and NT5, as well as their receptors including Trk A and Trk B, were increased. Under this scenario, the regulation of neurotransmitters and neurotropic factors could lead to a result of the improved behavior of those CMS-treated rats. However, the molecular targets of KXS in the brains have not been revealed, in particular this herbal mixture is containing numerical amount of chemicals [7].

 The four individual herbs of KXS are known to affect our nervous system and frequently applied in the treatment, or the prevention, of anti-depression, no matter in a form of single herb or in an herbal mixed formula [14]. In KXS, the four herbs could be separated into two herb pairs. The pair of Ginseng Radix et Rhizoma and Polygalae Radix is to invigorate the Xin-qi, while that of Acori Tatarinowii Rhizoma and Poria is to eliminate the dampness. The pharmacological effects of active chemicals deriving from these four herbs on anti-depression have been reported. The total saponins derived from Ginseng Radix et Rhizoma, especially ginsenosides, are the main chemicals with strong neurotrophic and neuroprotective effects [15]. Indeed, the saponins of Ginseng Radix et Rhizoma were shown to reverse the reduction in sucrose preference index in CMS-treated rats through enhancing the amount of monoamine neurotransmitter [16] and the expression of BDNF mRNA in hippocampus and frontal cortex of the brain [17]. In CMS-treated rats, the oligosaccharide ester of Polygalae Radix increased sucrose consumption, reduced the levels of cortistatin, adrenocorticotropic hormone, and corticotropin-releasing factor in serum, and also enhanced the expression of glucocorticoid receptor mRNA [18, 19]. The water extract of Acori Tatarinowii Rhizoma could significantly shortened the motionless time of forced swimming and the despair time of tail suspension in mouse animal models of depression [20]. Compared to other herbs, the studies of neuronal function of Poria are very limited, in spite of its widely application in treating mental disorder by herbalists. In line to its clinical usages, the water extract of Poria protected cultured PC12 cells through suppressing the oxidative stress and the apoptosis induced by A*β* [21]. The triterpenoids from Poria could regulate the expressed 5-HT3A receptors in *Xenopus* oocytes [10]. Although each herb showed the potent effect in anti-depression, a herbal mixed formula of four herbs was frequently applied clinically instead of a single form, according to the usages of Chinese medicine.

 The first catecholamine theory of depression was proposed in 1965 [22]. Thus, the aim of the drug development was to restore the decreased levels of neurotransmitters in the synaptic cleft or in the depressive brain. Dopamine, norepinepherine, and serotonin were regarded as the crucial neurotransmitters in the etiology of depression. Indeed, many drugs targeting to the neurotransmitter metabolism have been developed, for example, reserpine, tetrabenazine, iproniazid, and imipramine [4]. In the present studies, KXS treatment tended to restore the levels of dopamine, norepinepherine and serotonin in the CMS-treated rat brains: the restored levels were similar to that in the nonstressed group. Compared to the positive control imipramine, one of the first-generation tricyclic anti-depression drug and norepinepherine and serotonin reuptake inhibitors, KXS showed similar effects, which implied that it might exert anti-depression actions by modulating dopaminergic, noradrenalinergic, and serotoninergic neuronal systems.

**Figure 8:**
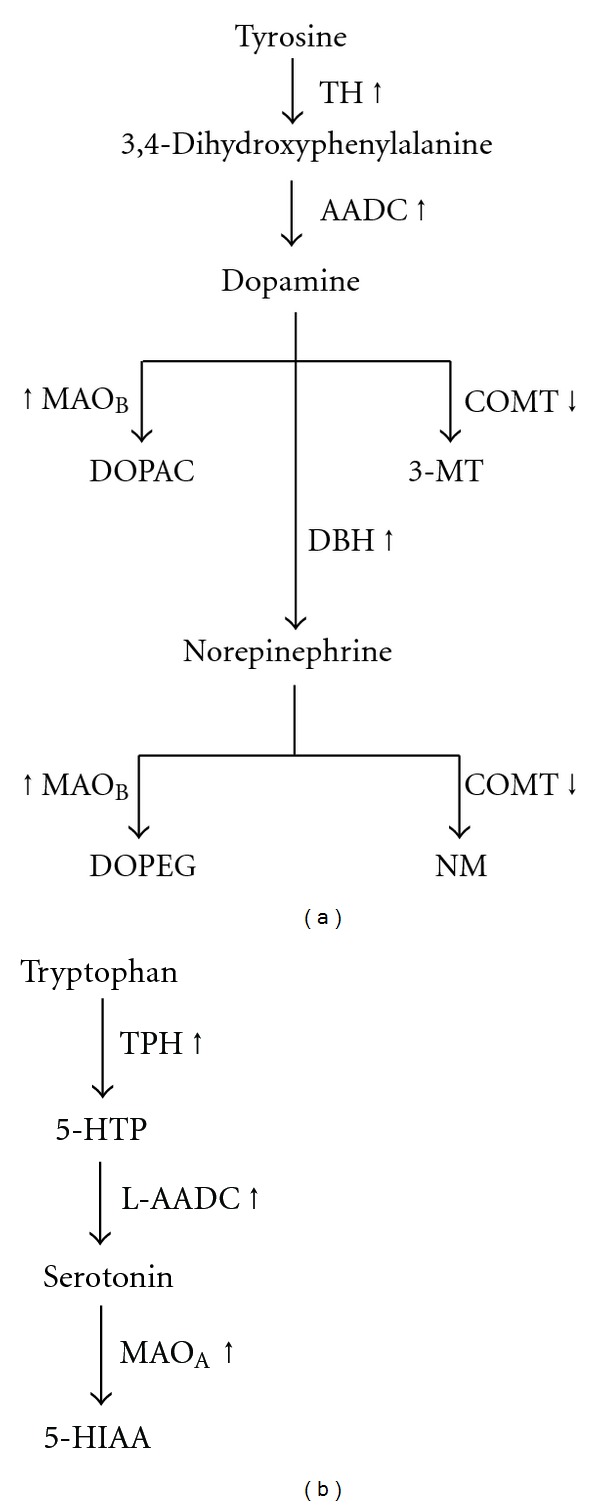
Synthesis and degradation of dopamine, norepinephrine, and serotonin. The detailed pathway of the synthesis and degradation of dopamine, norepinepherine (a) and serotonin (b) were shown. The arrows indicate the up- and downregulation of the corresponding mRNA level by the treatment of KXS. TH: tyrosine hydroxylase; AADC: aromatic acid decarboxylase; MAO_B_: monoamine oxidase B; COMT: catechol-O-methyl-transferase; DOPAC: dihydroxyphenylacetic acid; 3-MT: 3-methoxytyramine; DBH: dopamine *β*-hydroxylase; DOPEG: dihydroxyphenylglycol; NM: normetanephrine; TPH: tryptophan hydroxylase; 5-HTP: 5-hydroxytryptophan; MAO_A_: monoamine oxidase A; 5-HIAA: 5-hydroxyindole-3-acetic acid.

 In order to explore the mechanism of neurotransmitter regulation, the mRNA expression levels of related proteins were evaluated. For dopamine and norepinepherine, they share the same biosynthesis pathway at the start point, while that of serotonin has a distinct pathway (Figure 8). In the treatment of KXS in those CMS-treated rats, the enzymes responding for synthesis of dopamine/norepinephrine, and serotonin were increased, for example, tyrosine hydroxylase, dopamine *β*-hydroxylase, and aromatic acid decarboxylase. Under the CMS treatment, the brain receptors for dopamine and norepinepherine, and the transporter of serotonin were increased. Based on the results, KXS might regulate the neurotransmitter systems by acting on synthesis, storage, and upregulating the receptors in order to compensate for the deficient availability of neurotransmitters caused by the depressive state. Although imipramine could upregulate the levels of neurotransmitters, the regulations on those aforementioned proteins are very different to that of KXS treatment.

 The theory of neurotrophic factors in the etiology of depression has attracted much attention. The theory holds that the normal physiology of neuron is supported and maintained with the help of a series of neurotrophic factors, including NGF, BDNF, GDNF, NT-3, NT-4, and NT-5. If the deficiency of neurotrophic factors occurred, the neuron cannot survive and/or grow healthy, which will subsequently lead to neurodegenerative disorders, for example, depression. Postmortem analyses of brain tissues from patients with major depression showed a reduction in brain BDNF [23] and in serum BDNF [24, 25], whereas brain infusion of BDNF produced anti-depressant-like action in animals [26]. In addition, NGF was also found to have novel anti-depressant-like action in rats, but did not appear to have biochemical actions similar to that of other anti-depressants [27]. In the present study, the CMS-treated rat showed a decrease in the expression of neurotrophic factors and its corresponding receptors. However, KXS could increase the expression levels of neurotrophic factors and its receptors in restoring abnormal growth state of neuron under depression. Indeed, our preliminary results suggested that the application of KXS onto cultured astrocytes could induce the expression of neurotrophic factors (Zhu et al., unpublished results). For imipramine in CMS-treated rats, only the expression of GDNF level was elevated, which was also consistent with a previous report [28].

 Based on our current study, KXS might have multitargets in the brain for anti-depression. These findings are in line with previous studies. KXS treatment in rats has been shown to enhance the learning and memory abilities [29]. In parallel, KXS, in different ratio of the four herbs, was found to exert profound effects in anti-depression [16]. Thus, KXS could be a valuable herbal formula for the treatment of anti-depression, either as a form of health food supplement or a prescribed drug. More importantly, the toxicity of long-term intake of KXS has been very minimal, which has made it be used over a thousand of years in human [30].

## 5. Conclusions

 The treatment of KXS could alleviate the depression-like symptoms in CMS-induced rats. The anti-depressive action of KXS might be accounted by modulating the neurotransmitters system and increasing the expression of neurotrophic factors in the brain. This herbal extract is being chemically standardized and therefore could serve as alternative medicine or health food supplement for patients suffering from depression. Since KXS is a mixture of compounds, the identification of active ingredients here will be further evaluated.

## Supplementary Material

Supplementary Figure (A): Fingerprint chromatogram of KXS was made by HPLC-DAD at wavelength of 330 nm. The identification of 3, 6'-disinapoyl sucrose (1), **α**-asarone (7) and **β**-asarone (6) were shown in the chromatogram.
Supplementary Figure (B): Fingerprint chromatogram of KXS was made by HPLC-MS/MS method at negative scan mode. The identification of ginsenoside Rg_1_ (2), Re (3), Rb_1_ (4), Rd (5), and pachymic acid (8) were shown in the chromatogram. Representative chromatograms are shown, n=3.

## Figures and Tables

**Table 1:** Primer sequences, length of PCR products and optimal annealing temperature for each gene used in real-time quantitative PCR.

Primer	Sequence (5′-3′)	Source	bp	Ta (°C)
TH-S	CCA GTT CTC CCA GGA CAT TGG AC	NM_012740.3	312	59
TH-AS	GAG GCA TAG TTC CTG AGC TTG TCC			
DBH-S	GAA GAA TGC TGT GAC TGT CCA CCA G	NM_013158.2	387	59
DBH-AS	CAG AGG CTG CAG GTT CCA GTT AC			
AADC-S	GTT GTC ACC CTA GGA ACC ACA TCT TG	NM_012545.3	444	59
AADC-AS	CTC ATG AGA CAG CTT CAC GTG CTT TC			
MAO_A_-S	GCC AAA GTT CTG GGA TCT CAA GAA GC	NM_033653.1	204	59
MAO_A_-AS	CAC CAG TGA TCT TGA GCA GAC CAG			
MAO_B_-S	GAG AAG AAC TGG TGT GAG GAG CAG	NM_013198.1	342	59
MAO_B_-AS	AGC TGT TGC TGA CAA GAT GGT GGT			
COMT-S	GGT GAC GCG AAA GGC CAA ATC ATG	NM_012531.2	351	59
COMT-AS	CAG GCC ACA TTT CTC CAG GAG AAG			
DAT-S	GGT TCT ACG GCG TCC AGC AAT TC	M80570.1	291	59
DAT-AS	CAT AGG CCA GTT TCT CCC GGA AG			
VMAT2-S	GGT GGA CTC CTC TAT GAT GCC TAT C	NM_013031.3	351	59
VMAT2-AS	CTC CTT AGC AGG TGG ACT TCG AAG			
NET-S	CAG GTT CAG CAA TGA CAT CCA GCA G	NM_031343.1	282	59
NET-AS	GTG ATT CCG TAG GCC ACT CTC TC			
DrD2-S	AAC TGT ACC CAC CCT GAG GAC ATG	NM_012547.1	236	59
DrD2-AS	CTG TCA GGG TTG CTA TGT AGG CC			
Adra1A-S	TGG TGG GTT GCT TCG TCC TCT G	NM_017191.2	211	59
Adra1A-AS	CGA AGA CAC TGG ATT CGC AGG AC			
TPH-S	CAC CCA GGA TTC AAG GAC AAC GTC	NM_173839.2	421	59
TPH-AS	CAC TGT GAA GCC AGA TCG CTC TTT C			
SERT-S	ATG GTT CGT GCT CAT CGT GGT CAT C	NM_013034.3	268	59
SERT-AS	GAT GAA CAG GAG AAA CAG AGG GCT G			
Htr1A-S	CAT CAG CAA GGA CCA CGG CTA C	NM_012585.1	353	59
Htr1A-AS	GGA AGG TGC TCT TTG GAG TTG CC			
NGF-S	CAC TCT GAG GTG CAT AGC GTA ATG TC	XP_001067130.2	374	59
NGF-AS	CTG TGA GTC CTG TTG AAG GAG ATT GTA C			
BDNF-S	GAG CTG AGC GTG TGT GAC AGT ATT AG	BC087634	229	59
BDNF-AS	ATT GGG TAGT TCG GCA TTG CGA GTT C			
GDNF-S	GCG CTG ACC AGT GAC TCC AAT ATG	AF497634	318	59
GDNF-AS	CGC TTC ACA GGA ACC GCT ACA ATAT C			
NT3-S	ACA AGC TCT CCA AGC AGA TGG TAG ATG	M61179.1	310	59
NT3-AS	TCT CCT CGG TGA CTC TTA TGC TCT G			
NT4-S	TCA GTA CTT CTT CGA GAC GCG CTG	NM_013184.3	135	59
NT4-AS	GGC ACA TAG GAC TGT TTA GCC TTG CAT			
NT5-S	ATG CAG TGA GTG GCT GGG TGA C	S69323.1	229	59
NT5-AS	GTT TAG CCT TGC ATT CTG AGA GCC AG			
TrkA-S	ACC TCA ACC GTT TCC TCC GGT C	M85214.	330	57
TrkA-AS	CTC GAT CGC CTC AGT GTT GGA GA			
TrkB-S	CGG GAG CAT CTC TCG GTC TAT G	M55291.1	221	57
TrkB-AS	CAA ATG TGT CCG GCT TGA GCT GG			
TrkC-S	CAC TGT CTA CTA CCC TCC ACG TG	L03813.1	253	57
TrkC-AS	CTC TCT GGA AAG GGC TCC TTA AGG			
GAPDH-S	AAC GGA TTT GGC CGT ATT GG	Lee et al., 2009 [10]	516	57
GAPDH-AS	CTT CCC GTT CAG CTC TGG G			

Abbreviations: S: sense primer; AS: antisense primer; TH: tyrosine hydroxylase; DBH: dopamine *β*-hydroxylase; AADC: aromatic acid decarboxylase; MAO_A_: monoamine oxidase A; MAO_B_: monoamine oxidase B; COMT: catechol-O-methyltransferase; DAT: dopamine transporter; VMAT2: vesicular monoamine transporter 2; NET: norepinepherine transporter; DrD2: dopamine receptor D2; Adra1A: adrenergic receptor *α*1A; Htr 1A: serotonin receptor 1A; TPH: tryptophan hydroxylase; SERT: serotonin transporter; Htr1a: serotonin receptor 1A; NGF: nerve growth factor; BDNF: brain derived neurotrophic factor; GDNF: glial-cell-line-derived neurotrophic factor; NT3: neurotrophin 3; NT4: neurotrophin 4; NT5: neurotrophin 5; Trk A: tyrosine kinase receptor A; Trk B: tyrosine kinase receptor B; Trk C: tyrosine kinase receptor C; GAPDH: glyceraldehyde-3-phosphate dehydrogenase.
