# Supplementary material for: A Standardized Chinese Herbal Decoction, Kai-Xin-San, Restores Decreased Levels of Neurotransmitters and Neurotrophic Factors in the Brain of Chronic Stress-Induced Depressive Rats
Source: Evid Based Complement Alternat Med. 2012 Aug 29;2012:149256. doi: 10.1155/2012/149256 (PMC3437946; doi:10.1155/2012/149256)
Supplement: Supplementary file 1 — Supplementary Figure (A): Fingerprint chromatogram of KXS was made by HPLC-DAD at wavelength of 330 nm. The identification of 3, 6'-disinapoyl sucrose (1), α-asarone (7) and β-asarone (6) were shown in the chromatogram. Supplementary Figure (B): Fingerprint chromatogram of KXS was made by HPLC-MS/MS method at negative scan mode. The identification of ginsenoside Rg1 (2), Re (3), Rb1 (4), Rd (5), and pachymic acid (8) were shown in the chromatogram. Representative chromatograms are shown, n=3. [file 149256.f1.pdf]

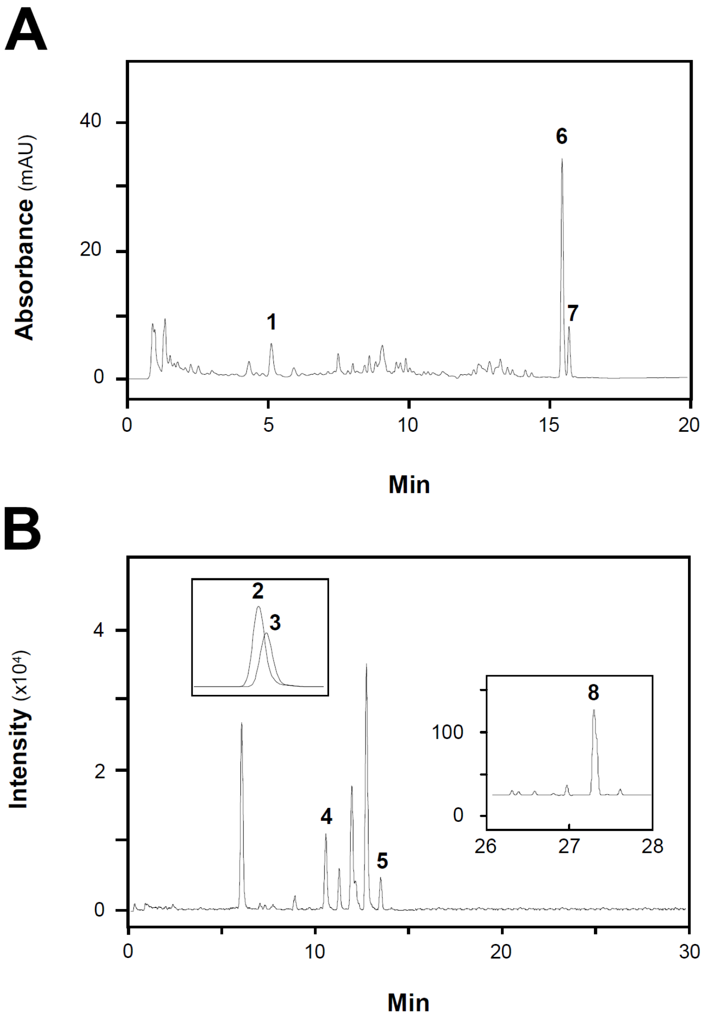

Supplementary Figure: Chemical fingerprint chromatograms of KXS.

(A): Fingerprint chromatogram of KXS was made by HPLC-DAD at wavelength of 330 nm. The identification of 3, 6'-disinapoyl sucrose (1), α-asarone (7) and β-asarone (6) were shown in the chromatogram. **(B):** Fingerprint chromatogram of KXS was made by HPLC-MS/MS method at negative scan mode. The identification of ginsenoside Rg_1 (2),

Re (3), Rb_1 (4), Rd (5), and pachymic acid (8) were shown in the chromatogram.

Representative chromatograms are shown, n=3.
